# The microbial production of kynurenic acid using *Yarrowia lipolytica* yeast growing on crude glycerol and soybean molasses

**DOI:** 10.3389/fbioe.2022.936137

**Published:** 2022-08-17

**Authors:** Magdalena Rakicka-Pustułka, Patrycja Ziuzia, Jan Pierwoła, Kacper Szymański, Magdalena Wróbel-Kwiatkowska, Zbigniew Lazar

**Affiliations:** Department of Biotechnology and Food Microbiology, Wrocław University of Environmental and Life Sciences, Wrocław, Poland

**Keywords:** kynurenic acid, *Yarrowia lipolytica*, yeast, tryptophan, soybean molasses, glycerol, non-conventional yeast

## Abstract

*Yarrowia lipolytica* yeast are able to produce kynurenic acid—a very valuable compound acting as a neuroprotective and antioxidant agent in humans. The recent data proved the existence of the kynurenine biosynthesis pathway in this yeast cells. Due to this fact, the aim of this work was to enhance kynurenic acid production using crude glycerol and soybean molasses as cheap and renewable carbon and nitrogen sources. The obtained results showed that *Y. lipolytica* GUT1 mutants are able to produce kynurenic acid in higher concentrations (from 4.5 mg dm^−3^ to 14.1 mg dm^−3^) than the parental strain (3.6 mg dm^−3^) in the supernatant in a medium with crude glycerol. Moreover, the addition of soybean molasses increased kynurenic acid production by using wild type and transformant strains. The A-101.1.31 GUT1/1 mutant strain produced 17.7 mg dm^−3^ of kynurenic acid in the supernatant during 150 h of the process and 576.7 mg kg^−1^ of kynurenic acid in dry yeast biomass. The presented work proves the great potential of microbial kynurenic acid production using waste feedstock. Yeast biomass obtained in this work is rich in protein, with a low content of lipid, and can be a healthy ingredient of animal and human diet.

## Introduction

**FIGURE 1:**
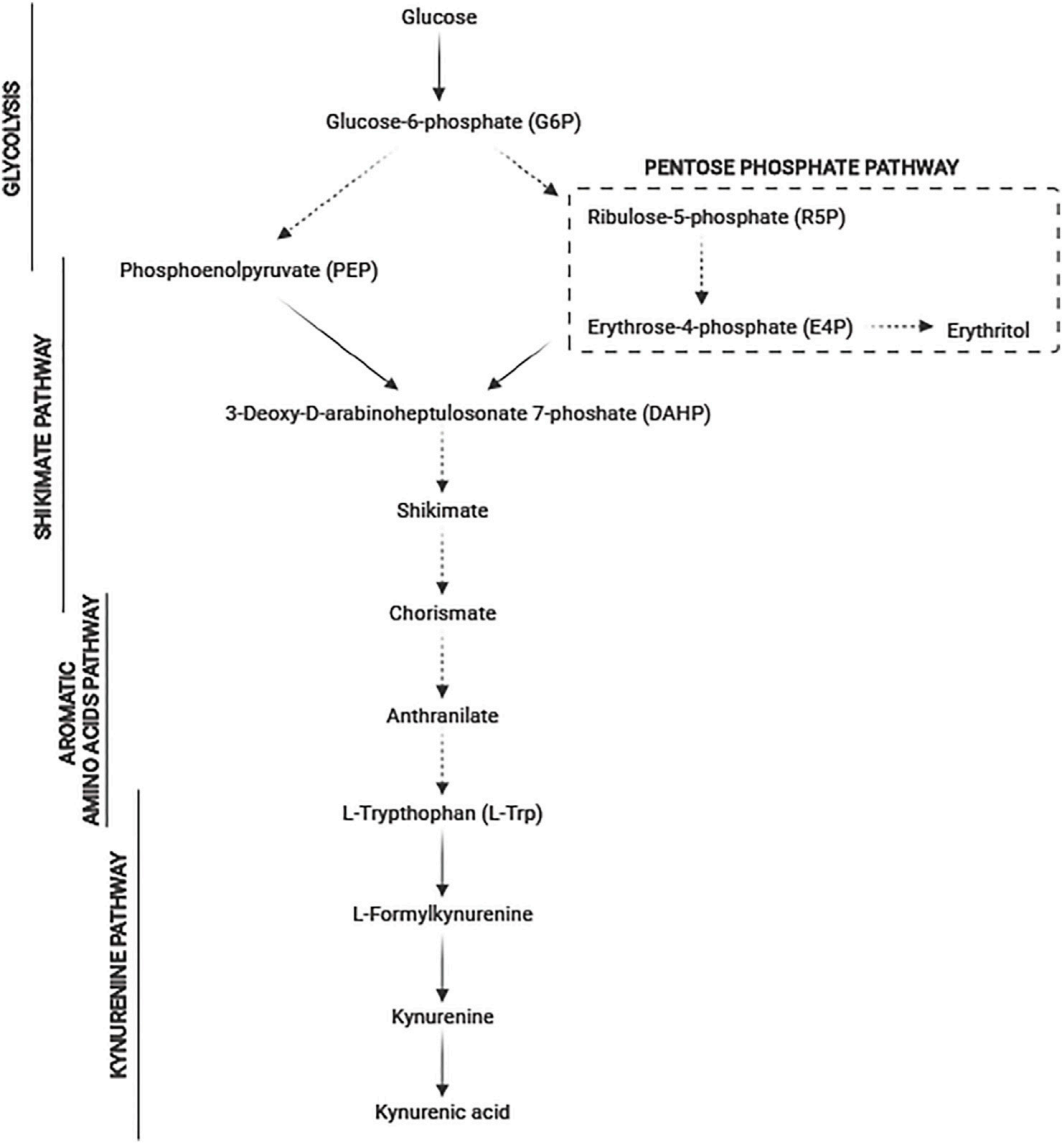
Metabolic pathway of tryptophan degradation to kynurenic acid in yeast (dashed line means multistage conversions; solid line means single-stage reactions).

Kynurenic acid (KYNA) plays a very important role in the human body due to its neuroprotective and antioxidant properties (Fuertig et al., 2016). KYNA is also credited with a role of N-methyl-D aspartate receptor antagonist and α7 nicotinic acetylcholine receptor antagonist (Lugo-Huitron et al., 2011). KYNA also shows its effects on AhR i GPR35 receptors. The negative feedback of these receptors regulates intestinal damage and inflammation to maintain intestinal integrity and homeostasis, through gradually sensing the KYNA level in the gut and macrophages (Wang et al., 2021). The disturbance in KYNA production is strongly correlated with various mental and neurodegenerative diseases (Erhardt et al., 2013; Singh et al., 2016). KYNA is a neuromodulator and controls the levels of glutamate, dopamine, acetylcholine, and α-aminobutyric acid. KYNA controls neuroendocrine functions and altered levels and is a potential marker in depression, schizophrenia, Alzheimer′s, and Huntington′s diseases. Normal levels of KYNA in the brain are crucial for the cognitive function (Ramos-Chávez et al., 2018). In bacteria, fungi, plants, and animals, KYNA is synthesized from tryptophan in the kynurenine pathway (Shin et al., 1991; Turski et al., 2011; Bortolotti et al., 2016) (Figure 1). The latest data showed the potential role of KYNA as a therapeutic agent for COVID-19 (Mei et al., 2021). Three active compounds from *Ephedra sinica* (4,6-dihydroxyquinoline-2-carboxylic acid, 4-hydroxyquinoline-2-carboxylic acid (KYNA), and 4-hydroxy-6-methoxyquinoline-2-carboxylic acid) were identified as being able to inhibit the interaction between angiotensin-converting enzyme 2 (ACE2) and the SARS-CoV-2 spike protein receptor-binding domain (SARS-CoV-2 RBD) (Mei et al., 2021). The recent data confirmed that the kynurenine pathway exists also in yeast cells (Figure 1). The enzymes of this pathway such as 3-hydroxyanthranilic acid dioxygenase and kynurenine aminotransferase were detected (Aro8 and Aro9) in *Saccharomyces cerevisiae* (Ohashi et al., 2017). Furthermore, our previous studies confirmed the possibility of KYNA synthesis by the nonconventional yeast *Yarrowia lipolytica* (Wróbel-Kwiatkowska et al., 2020a). The KYNA is now industrially produced *via* chemical synthesis. In our opinion, finding an efficient producer microorganism is reasonable. Interestingly, KYNA was detected in plant-based foods, honey samples, fermented foods (beer, wine, mead, and spirits), and herbs (Turski et al., 2012; Turski et al., 2015; Turski et al., 2016; Yilmaz and Gokmen, 2018). However, low concentration of KYNA in natural sources and its low aqueous solubility limit the therapeutic activity of KYNA and its application (Dhakar et al., 2019). The latest research reported that intraperitoneal administration of KYNA prevented high-fat diet–induced body weight gain in mice (Li et al., 2018). Moreover, in another research, KYNA in a concentration of 5 mg kg^−1^ day^−1^, inhibited the body weight gain and the increase of average daily energy intake in mice (Li et al., 2021). Moreover, the authors also presented the positive possible effects of KYNA on gut microbiota in high-fat diet mice (Li et al., 2021). In the literature, only few microorganisms producing KYNA are characterized, and all of them are yeast. *S. cerevisiae* is able to produce KYNA in a concentration of 9.146 mg dm^−3^ growing on glucose and 2.36 mg dm^−3^ growing on ground malt (Yılmaz and Gökmen, 2019; Cas et al., 2021). Quite low KYNA production (0.236 mg dm^−3^) was also noted for *S. pastorianus* on ground malt (Yılmaz and Gökmen, 2019). Up to now, the highest reported concentration of KYNA was achieved by *Y. lipolytica* S12 growing on chestnut honey (68 mg dm^−3^) and fructose (21.38 mg dm^−3^) (Wróbel–Kwaitkowska et al., 2020a; Wróbel–Kwaitkowska et al., 2020b).

As it was already mentioned, a promising KYNA producer may be the *Y. lipolytica* yeast. This species is well known for its abilities to use cheap and renewable carbon sources, such as glycerol. One of the processes in which raw glycerol is generated in high quantities is biodiesel production from rapeseed oil. Glycerol can be used as the source of carbon with a great variety of applications in industries (food industry, chemical industry, polymer industry, personal care products, therapeutic, and diagnostic applications) (Mota et al., 2017). However, some impurities present in crude glycerol may hinder its functional applications. Therefore, the biotechnological process, in which the direct utilization of crude glycerol is possible, is very desirable. Up to now, crude glycerol was used as a substrate in citric acid, erythritol, α-ketoglutaric acid, lipids, and protein production by *Y. lipolytica* (Dobrowolski et al., 2016; Cybulski et al., 2018; Qian et al., 2020; Rakicka-Pustułka et al., 2020). However, crude glycerol was never applied for KYNA biosynthesis by this yeast. Furthermore, due to the fact that KYNA biosynthesis follows the tryptophan degradation pathway, another raw material rich in proteins may be used as a source of this amino acid. Such a substrate may include soybean molasses, pumpkin pomace, evening primrose pomace, and sunflower pomace. The soybean molasses is formed during the production of soy protein concentrates and isolates (Siqueira et al., 2008). A typical soybean manufacturing company produces 0.36 ton of molasses per ton of the soy protein concentrate (Siqueira et al., 2008). The soybean molasses is the raw material rich in protein (peptides and amino acids), carbohydrates (sucrose, stachyose, and raffinose), lipids, and other macro- and micro-nutrients which can support microbial growth and bio-product synthesis (Cheng et al., 2017). The polysaccharides present in soybean molasses are, in most cases, inaccessible to microorganisms due to lack of enzymes required for their hydrolysis. Therefore, soy molasses currently has limited use, especially as animal feed, however, with a very low value (Loman and Kwang Ju, 2017). Some other applications of this substrate include butanol (Dong et al., 2014), ethanol (Romão et al., 2012), sophorolipid (Solaiman et al., 2004), and polyhydroxyalkanoate (Solaiman et al., 2006) production.

Due to the attractive biological properties of KYNA and the unexplored potential of particular strains of *Y. lipolytica* for its biosynthesis, the aim of this work was to enhance KYNA production using crude glycerol and soybean molasses as cheap and renewable carbon and nitrogen sources. Furthermore, *Y. lipolytica* A101-1.31 and its eight derivatives (A 101-1.31 GUT1/1 to A 101-1.31 GUT1/8) with an overexpression of glycerol kinase (*GUT1*) under the control of the strong, constitutive *TEF* promoter were used to enhance glycerol consumption. The mutants were obtained by random integration in the genome of the (*GUT1*) expression cassette. Our findings further indicate the potential role of KYNA and the biomass of *Y. lipolytica* as a functional food ingredient useful in the treatment of obesity and hyperlipidemia, as well as for the modulation of gut microbiota in animals and humans.

## Material and methods

### Strains

Strains used in this study are presented in Table 1. The acetate-negative mutant strain *Y. lipolytica* A-101.1.31 and eight recombinant strains of *Y. lipolytica*—A-101.1.31 GUT1/1, A-101.1.31 GUT1/2, A-101.1.31 GUT1/3, A-101.1.31 GUT1/4, A-101.1.31 GUT1/5, A-101.1.31 GUT1/6, A-101.1.31 GUT1/7, and A-101.1.31 GUT1/8—were used. *Y. lipolytica* A-101.1.31 was used as a control strain. All strains belong to the Department of Biotechnology and Food Microbiology, Wroclaw University of Environmental and Life Sciences, Poland. The construction of recombinant strains was previously described in Tomaszewska-Hetman et al. (2021). The yeast strains were stored in glycerol solution 25% (v v^−1^) at −80°C. Before use, YPD plates were inoculated and incubated for 24 h at 28°C to refresh the yeast biomass.

**TABLE 1:** Strains used in this study.

Strain	Plasmid, genotype	Reference
*Yarrowia lipolytica* strains		
A-101.1.31	Wild type, an acetate-negative mutant, and uracil prototroph	Rywińska et al. (2003)
A-101.1.31 GUT 1/1	TEF-*GUT 1*	Tomaszewska-Hetman et al. (2021); this study
A-101.1.31 GUT 1/2	TEF-*GUT 1*	Tomaszewska-Hetman et al. (2021); this study
A-101.1.31 GUT 1/3	TEF-*GUT 1*	Tomaszewska-Hetman et al. (2021); this study
A-101.1.31 GUT 1/4	TEF-*GUT 1*	Tomaszewska-Hetman et al. (2021); this study
A-101.1.31 GUT 1/5	TEF-*GUT 1*	Tomaszewska-Hetman et al. (2021); this study
A-101.1.31 GUT 1/6	TEF-*GUT 1*	Tomaszewska-Hetman et al. (2021); this study
A-101.1.31 GUT 1/7	TEF-*GUT 1*	Tomaszewska-Hetman et al. (2021); this study
A-101.1.31 GUT 1/8	TEF-*GUT 1*	Tomaszewska-Hetman et al. (2021); this study

### Substrate

Crude glycerol from the biodiesel industry with the purity of 870 g kg^−1^ (Wratislavia-Bio, Wroclaw, Poland) was used as a source of carbon and energy. Crude glycerol consisted of water—15% (m/m), sodium chloride—4.3% (m/m), matter organic non glycerol MONG—6% (m/m), methanol—0.3% (m/m), nitrogen content—0.023, ash—0.93 and metals Cu—0.096 mg/kg, Fe—1.99 mg/kg, and Zn—0.09 mg/kg. Soybean molasses (Sojaprotein a. d. Bečej, Serbia) was used as a source of nitrogen and tryptophan—the precursor of synthesis of kynurenic acid in yeast cells. The composition of the soybean molasses is presented in Table 2.

**TABLE 2:** Composition of soybean molasses used in the presented study.

Compound	Amount
Total carbohydrates (%)	33.00
Protein (%)	6.84
Lipids (%)	6.12
Ash (%)	5.54
Dry weight (%)	72.45
Amino acids (g kg^−1^)	
Asp	8.26
Thr	0.97
Ser	1.3
Glu	8.09
Pro	1.16
Gly	1.18
Ala	2.83
Val	0.87
Ile	0.7
Leu	1.08
Tyr	0.86
Phe	1.61
His	2.3
Lys	0.75
Arg	2.04
Trp	1.1
CysH	1.25
metS	0.59

### Media and culture conditions

The rich YPG medium was used for the yeast inoculum preparation. The composition of medium is included in Table 3. The inoculum was grown in 50 cm^3^ YPG medium for 48 h in 250-cm^3^ flasks on a rotary shaker at 28°C and 140 rpm. The precultures accounted for 10% of the production culture working volume, and the initial optical density (OD_600_) was adjusted to 0.5.

**TABLE 3:** Composition of media used in this study.

Media	Composition	Concentration (g dm^−3^)	Purpose
YPG	Yeast extract (Merck, Germany)	10	Yeast inoculum preparation
	Peptone (Biocorp, Poland)	10	
	Technical grade glycerol (POCH, Gliwice, Poland)	20	
Crude glycerol medium	Crude glycerol	40	Kynurenic acid production of mutants in bioreactor cultures Wróbel-Kwiatkowska et al. (2020a)
	(NH_4_)_2_SO_4_	9	
	MgSO_4_ × 7 H_2_O	0.3	
	KH_2_PO_4_	0.25	
	Yeast extract (Merck, Germany)	1	
Soybean molasses medium	Soybean molasses	60	Kynurenic acid production of mutants in bioreactor cultures
	Crude glycerol	40	
	(NH_4_)_2_SO_4_	9	
	MgSO_4_ × 7 H_2_O	0.3	
	KH_2_PO_4_	0.25	
	Yeast extract (Merck, Germany)	1	

### Bioreactor studies

Kynurenic acid production by *Y. lipolytica* transformants and the control WT strain was analyzed in bioreactor cultures with crude glycerol in medium with the composition listed in Table 3. The C:N ratio of the production medium with crude glycerol was 9:1. The effect of the precursor tryptophan present in soybean molasses was tested in the medium described in Table 3. The medium was supplemented with soybean molasses solution. The C:N ratio of medium supplemented with soybean molasses was around 11:1. Here, 50% (w/v) of the solution of soybean molasses in water was prepared and added to the bioreactor in four portions (60 cm^3^ each) at 24, 48, 72, and 96 h of cultivation. The final concentration of tryptophan added to the culture reached 27.5 mg dm^−3^. All bioreactor cultures were performed in a 5-dm^3^ stirred-tank reactor (Biostat B Plus, Sartorius, Germany) with the working volume of 1.6 dm^3^ at 28°C. Aeration and stirring rates were set at 0.8 vvm and 800 rpm, respectively. pH 5.4 was maintained automatically by the addition of 20% (w/v) NaOH solution during the first 24 h of the process. After that, 10% HCl solution (v/v) was used to maintain the pH at 5.4. All cultures were cultivated for 168 h. Before the run, all bioreactors with the appropriate medium were sterilized in an autoclave at 121°C for 20 min. All cultures were conducted in three biological replicates, and standard deviations were calculated.

### Analytical methods

Here, 10 cm^3^ of culture broth was centrifuged at 6,000 g. The biomass was washed twice with distilled water, harvested by filtration on 0.45-µm pore-size membranes, and air-dried for 24 h. The biomass concentration was determined gravimetrically after drying at 105°C and expressed in grams of cell dry weight per dm^3^ (g dm^−3^). The concentration of glycerol was analyzed by high-performance liquid chromatography (Dionex-Thermo Fisher Scientific, United Kingdom) with a HyperRez Carbohydrate H+ column (Thermo Scientific, Waltham, MA). The column was eluted with 25 mM trifluoroacetic acetic acid with a rate of 0.6 cm^3^ min^−1^ and coupled to a UV (k = 210 nm) and a refractive index (RI) detector (Shodex, Ogimachi, Japan). Kynurenic acid was measured using a high-performance liquid chromatography system (Dionex-Thermo Fisher Scientific, United Kingdom) equipped with an ultra-sensitive diode array detector (Dionex-Thermo Fisher Scientific, United Kingdom) (k = 286 nm) and a Hypersil GOLD™ C18 column (150 × 4.6 mm, 3 μm), maintained at 30°C. The isocratic mobile phase of 15 mM potassium phosphate (pH 6.4), with 2.7% (v/v) acetonitrile was used with a flow rate of 1.2 cm^3^ min^−1^. The run time was set for 15 min. External standards (Merck, Germany) were applied for identification and quantification of peak areas. Kynurenic acid was also measured in the dry biomass, and the procedure for extraction was as follows. Initially, 10 cm^3^ of the culture at the end of the process was centrifuged (6,000 g, 5 min), and the yeast biomass, without any washing steps, was dried at room temperature for 48 h. In the next step, the dried biomass was ground in a biomass grinder and mixed with water in 15-cm^3^ falcon tube (1:7 (w/w)). The sample was kept at room temperature for 1 h, followed by vortexing for 5 min and centrifugation (6,000 g, 5 min). After that, KYNA concentration was quantified in the supernatant using the HPLC method described earlier.

Additionally, changes in the protein concentration in the yeast biomass during the culture were investigated. For that purpose, samples of the culture broth (200 cm^3^) were withdrawn from the bioreactor at 24 h and at the end of the culture. After centrifugation (6,000 g, 5 min), the yeast biomass was washed twice with distilled water and air dried. The protein content was determined in the dry biomass using the Kjeldahl method (Kjeldahl, 1883). Furthermore, the lipid concentration in the obtained biomass was determined using the methodology described previously by Rakicka-Pustułka et al. (2021).

### Genome walking

To analyze the site of *GUT1* gene containing expression cassette integration into the *Y. lipolytica* A101-1.31 genome, the Universal GenomeWalker™ 2.0 kit (Clontech Laboratories, Inc., TaKaRa) was used, according to the manufacturer’s instruction. The amplified PCR fragments were cloned into the pJET1.2/blunt cloning vector (CloneJET PCR Cloning Kit, Thermo Scientific) and sent for sequencing. The obtained results were analyzed using the BLAST algorithm available on the gryc.inra.fr website.

## Results

### Kynurenic acid production in the bioreactor culture on crude glycerol

**FIGURE 2:**
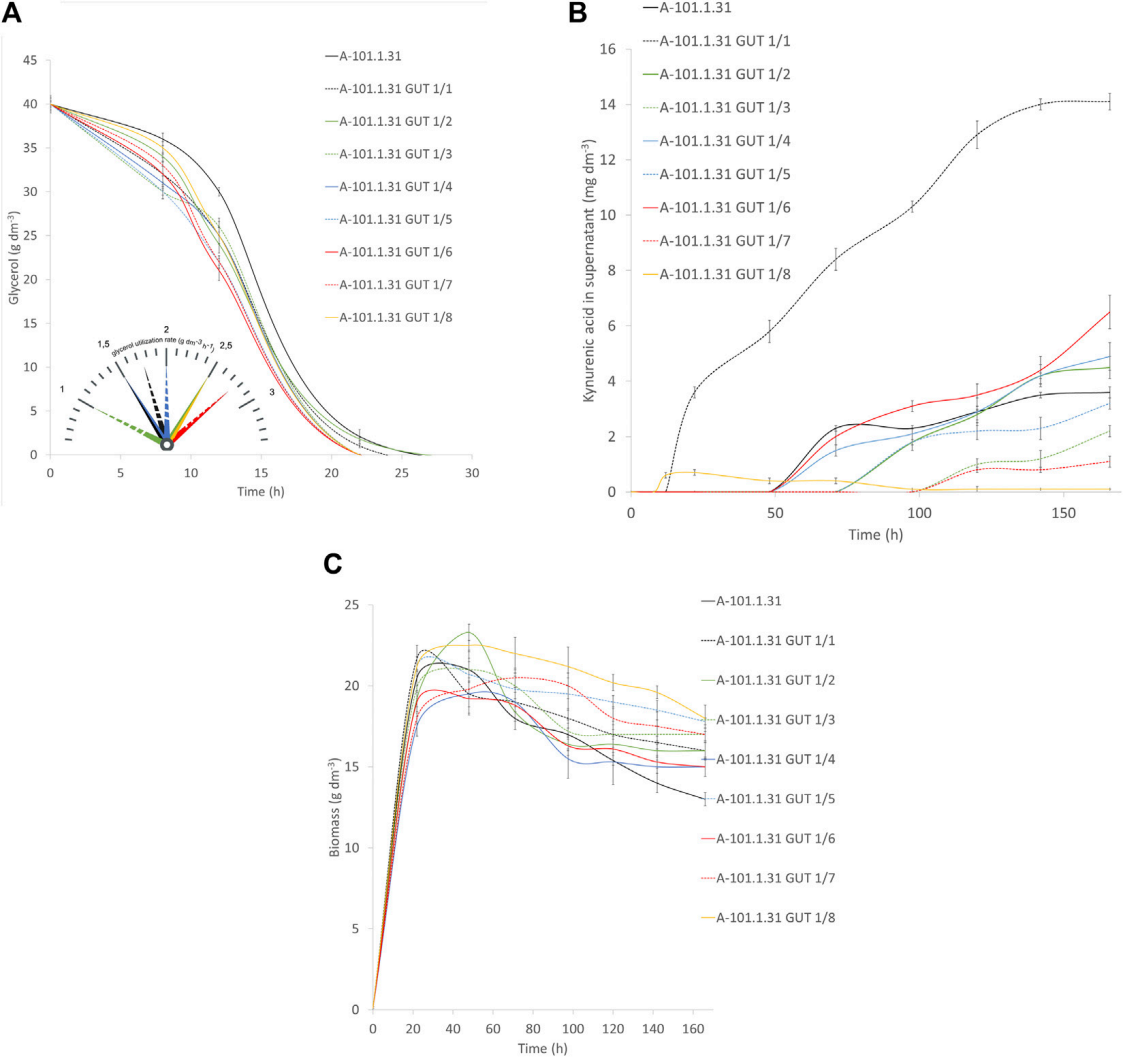
Waste glycerol utilization **(A)** and kynurenic acid **(B)** and biomass **(C)** production during batch bioreactor cultures of the *Y. lipolytica* A-101.1.31 strain and mutant strains of *Y. lipolytica* (A-101.1.31 GUT 1/1—A-101.1.31 GUT1/8) overexpressing glycerol kinase. In the Panel A, the glycerol utilization rate for mutants is presented in the time between 8 and 12 h.

**FIGURE 3:**
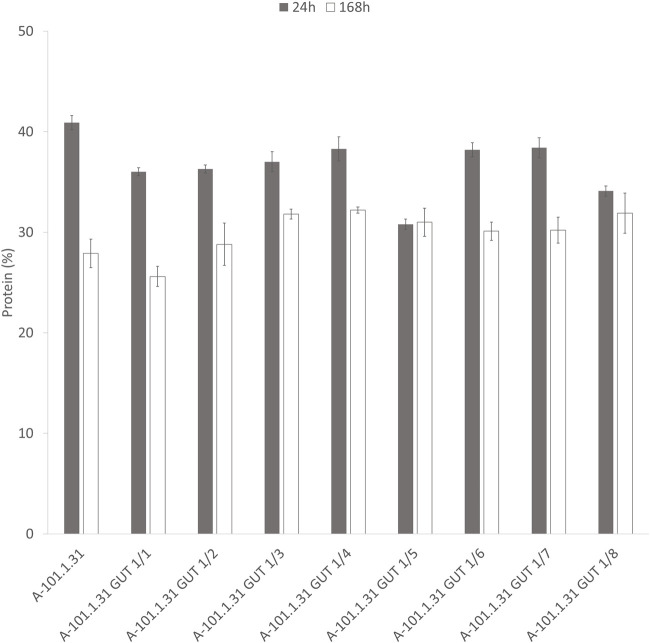
Protein content of *Y. lipolytica* biomass (WT and *GUT1* transformants) during kynurenic acid production in batch bioreactor cultures on waste glycerol (■) 24 h of the culture and (□) 168 h of the culture.

**FIGURE 4:**
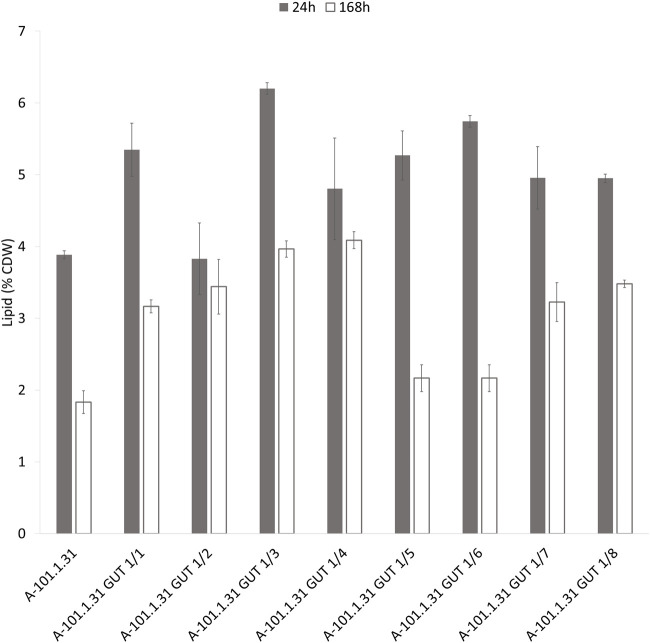
Lipid content of *Y. lipolytica* biomass (WT and *GUT1* transformants) during kynurenic acid production in batch bioreactor cultures on waste glycerol (■) 24 h of the culture and (□) 168 h of the culture.

Eight strains of *Y. lipolytica* with the overexpression of glycerol kinase (*GUT1*) were investigated for the production of KYNA from crude glycerol derived from the biodiesel industry. The medium used for the KYNA production was previously tested for a specific S12 *Y. lipolytica* strain (Wróbel-Kwiatkowska et al., 2020a). In Figure 2A, the glycerol consumption by the tested strains is presented. Seven mutants used glycerol faster than the control WT strain (*Y. lipolytica* A-101.1.31). The glycerol utilization rate between 8 and 12 h was the highest for A-101.1.31 GUT1/6 and GUT 1/7 (2.75 g dm^−3^ h^−1^), while for the WT strain, it was 1.5 g dm^−3^ h^−1^ (Figure 2A). The transformants produced KYNA in the concentration ranging from 0.1 to 14.1 mg dm^−3^ (Figure 2B). The highest concentration of KYNA in the fermentation broth was observed for the A-101.1.31 GUT1/1 strain. Furthermore, the concentration of KYNA in the yeast dry biomass at the end of the culture was also investigated (Table 4). The highest amount of KYNA was noted for the A-1-1.1.31 GUT1/1 transformant (357.9 mg kg^−1^ CDW). The biomass concentration increased during the first 20–24 h, and after glycerol was exhausted, its concentration decreased, however, with a different dynamics for every strain (Figure 2C). At the end of the culture, the highest biomass was noted for A-101.1.31 GUT1/5 and A-101.1.31 GUT1/8 mutants (17.8 and 18 g dm^−3^, respectively) and the lowest biomass for the control strain A-101.1.31 (13 g dm^−3^). The protein and lipid concentrations in the yeast biomass are presented in Figures 3, 4. The protein content after 24 h of the culture was high for all analyzed strains (between 30 and 40%) and decreased at the end of the process (Figure 3). Interestingly, the lowest protein content after 168 h was observed for the best KYNA producer A-101.1.31 GUT 1/1. The nitrogen-rich medium used in this study did not favor lipid production by this yeast. The concentration of lipids did not exceed 6% of CDW after 24 h of the culture and similarly to protein level, after glycerol exhaustion decreased in all strains (Figure 4).

**TABLE 4:** Kynurenic acid in dry biomass of *Yarrowia lipolytica* strains growing on crude glycerol.

Strain	A-101.1.31	A-101.1.31 GUT 1/1	A-101.1.31 GUT 1/2	A-101.1.31 GUT 1/3	A-101.1.31 GUT 1/4	A-101.1.31 GUT 1/5	A-101.1.31 GUT 1/6	A-101.1.31 GUT 1/7	A-101.1.31 GUT 1/8
KYNA in dry biomass (mg kg^−1^ CDW)	31.4 ± 0.5	357.9 ± 1.3	115.6 ± 0.9	133.7 ± 0.5	57.6 ± 0.3	149.4 ± 1.0	117.5 ± 0.4	129.9 ± 0.7	50.0 ± 0.3

### Kynurenic acid production from glycerol and soybean molasses medium

**FIGURE 5:**
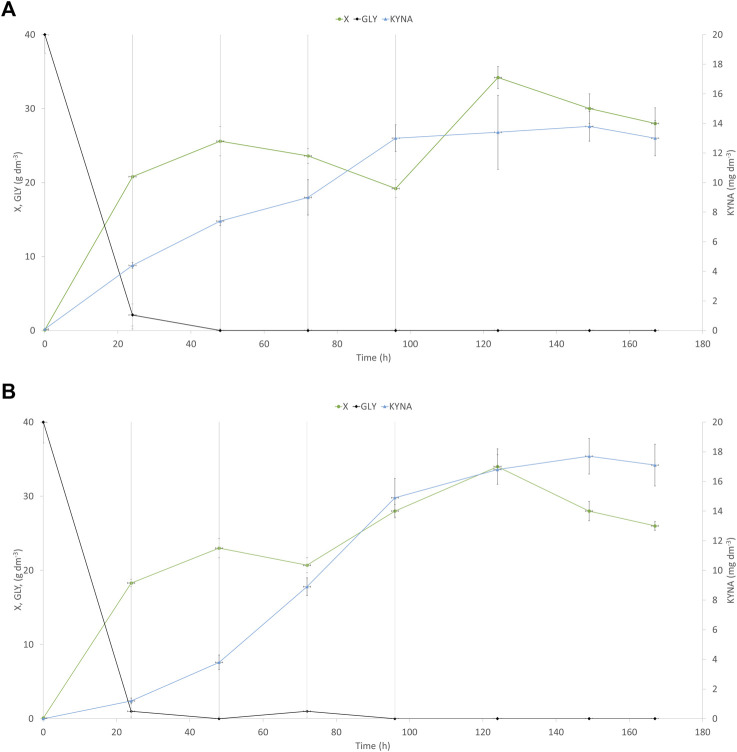
Kinetics of yeast growth (●), glycerol consumption (♦), and kynurenic acid production (▲) of the *Y. lipolytica* A-101.1.31 strain **(A)** and mutant strain of *Y. lipolytica* A-101.1.31 GUT 1/1 **(B)** growing on waste glycerol and soybean molasses added in four portions (indicated as gray vertical lines). X-biomass; GLY-glycerol; KYNA-kynurenic acid.

**FIGURE 6:**
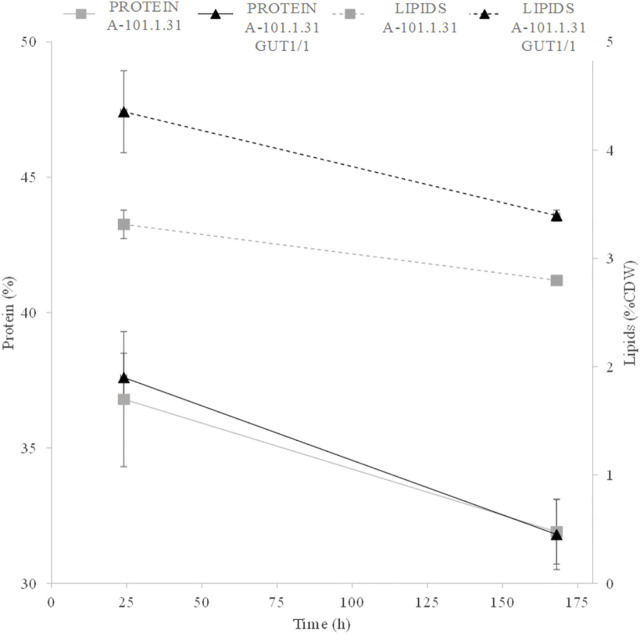
Protein (solid line) and lipid (dotted line) contents during kynurenic acid production by the *Y. lipolytica* A-101.1.31 (■) strain and mutant *Y. lipolytica* A-101.1.31 GUT 1/1 strain (▲) growing on waste glycerol and soybean molasses.

In the next step, to enhance KYNA production, fed-batch cultures with soybean molasses supplementation were used. One *Y. lipolytica* strain—the best KYNA producer using waste glycerol—was chosen for further investigation (A-101.1.31 GUT1/1). The process began with raw glycerol as a substrate, and after 24, 48, 72, and 96 h of the culture, four portions of soybean molasses (50% w v^−1^) were added to the bioreactor (60 cm^3^ each). The final concentration of tryptophan added to the medium reached 27.5 mg dm^−3^. Soybean molasses used in this study was rich in tryptophan (55 mg 100 g^−1^)—a precursor of KYNA synthesis in yeast. Indeed, the addition of soybean molasses increased KYNA production by both analyzed strains (WT and the best KYNA producer) (Figure 5). The control strain (Figure 5A) produced almost 4-times higher amount of KYNA (13.8 mg dm^−3^) after 150 h of the process than the process without supplementation. At the same time, the A-101.1.31 GUT1/1 mutant strain produced 17.7 mg dm^−3^ of KYNA during 150 h of the process, which was only 25% higher than the process without tryptophan supplementation (Figure 5B). For both strains, the concentration of KYNA decreased slightly in the late stage of the process (Figures 5A,B). The concentration of dry biomass for both strains decreased at the end of the process; however, the final concentration was much higher (26–28 g dm^−3^ for both strains; Figures 5A,B) than the process without soybean molasses supplementation. The obtained yeast biomass was also rich in KYNA (Table 5). The A-101.1.31 GUT 1/1 strain produced 576.7 mg kg^−1^ CDW of KYNA in dry biomass and was 46% higher than that for the control strain (Table 5). Similar to the previous process, the protein and lipid contents in the biomass were also analyzed (Figure 5). The biomass after soybean molasses supplementation was rich in proteins (>30% of CDW for both strains). Furthermore, during the whole process, in addition to high KYNA production, the protein concentration decreased only about 5% (Figure 6), which is a much better result than the batch cultures without soybean molasses addition (Figure 3). Additionally, the obtained yeast biomass contained low amount of lipids (Figure 6), which did not exceed 5% of CDW after 24 h and at the end of the process reached about 3% for both strains (Figure 6).

**TABLE 5:** Comparison of kynurenic acid (KYNA) production by various strains.

Strain	Substrate	Tryptophan supplementation (mg dm^−3^)	KYNA	Reference
*Yarrowia lipolytica* S12	Fructose	200	21.38 mg dm^−3^ ^a^/494.16 mg kg^−1^ ^b^	Wróbel-Kwiatkowska et al. (2020a)
*Yarrowia lipolytica* S12	Chestnut honey	—	68 mg dm^−3^ ^a^/542 mg kg^−1^ ^b^	Wróbel-Kwiatkowska et al. (2020b)
*Saccharomyces cerevisiae* EC1118	Glucose	400	9.146 mg dm^−3^	Cas et al. (2021)
*Saccharomyces cerevisiae* NCYC 88	Ground malt	300	2.36 mg dm^−3^	Yılmaz and Gökmen (2019)
*Saccharomyces pastorianus* NCYC 203	Ground malt	300	0.236 mg dm^−3^	Yılmaz and Gökmen (2019)
*Yarrowia lipolytica*	Waste glycerol and soybean molasses	27.5	17.7 mg dm^−3^ ^a^	This work
A-101.1.31 GUT 1/1			576.7 mg kg^−1^ ^b^	
*Yarrowia lipolytica*			13.8 mg dm^−3^	
A-101.1.31			394.5 ± 1.8 mg kg^−1^ ^b^	

a Concentration in the supernatant.

b Concentration in dry yeast biomass.

### Analysis of the integration site of the glycerol kinase gene

To understand the performance of the A-101.1.31 GUT 1/1 strain in terms of KYNA production, the site of *GUT1* gene integration into the *Y. lipolytica* genome was analyzed. The expression cassette was composed of partial ZETA sequences (retrotransposable elements) bordering the expression cassette and the selection marker. These sequences help in random chromosomal integration of the inserting DNA using the non-homologous end joining pathway. Using the Universal GenomeWalker™ 2.0 kit and following the manufacturers instruction, two copies of the *GUT1* expression cassette were identified in the analyzed transformant. Both affected genes are on the chromosome E of *Y. lipolytica* (YALI0E24453g and YALI0E32835g). YALI0E24453g encodes a protein with some similarities with uniprot|P40344 *S. cerevisiae* YNR007c *AUT1* that is essential for autophagocytosis, whereas YALI0E32835g encodes *POX1* fatty-acyl coenzyme A oxidase.

## Discussion

The knowledge and possibility of KYNA production using yeast is currently of growing interests. So far, KYNA was identified in *S. cerevisiae, S. pastorianus*, and *Y. lipolytica* yeast species (Table 5). The substrates used for KYNA production during microbial fermentation were glucose, fructose, honey, and ground malt (Yılmaz and Gökmen, 2019; Wróbel-Kwiatkowska, et al., 2020a; Cas et al., 2021). Some of the performed batch cultures presented in Table 5 were supplemented with pure tryptophan in different concentrations (from 200 to 400 mg dm^−3^). Up to now, the highest concentration of KYNA in the post culture medium was noted for the *Y. lipolytica* S12 strain growing on chestnut honey (68 mg dm^−3^) (Wróbel-Kwiatkowska et al., 2020b). However, utilization of such a valuable product as honey for microbial fermentation can be controversial and unprofitable. Nowadays, it is required that the modern biotechnology fulfills the principles of circular economy and converts feedstock into value-added chemicals (Hernández-Pérez et al., 2020). Due to this fact, in the presented work, crude glycerol obtained from the biodiesel industry was chosen as a substrate for KYNA production using *Y. lipolytica* as a microbial factor for its biosynthesis. It is also worth to point out that the global increasing production of biodiesel delivers annually high amount of crude glycerol (Johnson and Taconi, 2007; Rakicka-Pustułka et al., 2021). The possibility to use this feedstock without any expensive purification steps for KYNA biosynthesis is a huge advantage of the presented process. Using *Y. lipolytica* and glycerol in one process of KYNA production fulfills the main principles of cleaner production, energy saving potential, and sustainable development. Furthermore, our strains were improved for better glycerol utilization by overexpression of the glycerol kinase (*GUT1*) under the control of the strong, constitutive TEF promoter. This modification was previously described in other *Y. lipolytica* strains to improve glycerol assimilation (Mirończuk et al., 2016).

From several initial cultures preformed during screening for the best *Y. lipolytica* producer (data not shown), it is now known that KYNA does not accumulate inside the yeast cells. Most of this compound is secreted to the culture medium, sticks to the cell wall in some way on the outside of the cells, and concentrates during biomass drying. Only after extensive biomass washing with water, the concentration of KYNA drastically decreases in the dry biomass. Applying the harsh washing step in the presented work allowed to be rich as much as 576.7 mg kg^−1^ CDW in the supernatant (after samples being combined) for the *Y. lipolytica* A-101.1.31 GUT 1/1 transformant using crude glycerol as a carbon source. This is the highest value described so far in the literature for microbial KYNA production.

The fact that not much information is available of microbial utilization of soybean molasses in biotechnological processes is also important. One of the reasons of the occasional utilization of soybean molasses in fermentation processes is the large amounts of carbohydrates such as stachyose, raffinose, and sucrose present in this feedstock, which cannot be consumed by microorganisms (de Oliveira et al., 2020). However, finding new ways to manage this food-based matrix naturally rich in proteins seems warranted (Table 2). In our current study, we proved that the soybean molasses can be used as a cheap natural source of tryptophan for KYNA production. The positive effect on the increase in KYNA production was particularly evident for the control strain; however, a smaller increase was also observed for the *Y. lipolytica* A-101.1.31 GUT 1/1 transformant. The use of soybean molasses eliminated the necessity for addition of pure tryptophan to the medium. What is even more beneficial is that the utilization of the soybean molasses improved significantly the production of yeast biomass, which could be further used as a supplement of functional food. Cas et al. (2021) have recently presented that soybean flour may be used as a carbon source for KYNA production. Moreover, Vitalini et al., 2020 also demonstrated that toasted soybean flour treated by water extraction at room temperature allows to obtain free tryptophan, potentially bioavailable for yeast during fermentation as a precursor of KYNA. However, utilization of such a valuable product as flour for microbial KYNA production can be controversial and unprofitable. It is commonly known that several raw materials fermented throughout spontaneous or controlled processes have beneficial effects on human health (Sanlier et al., 2019). Furthermore, yeasts share with higher eukaryotes several metabolic pathways, which are relevant reservoirs of functional molecules, such as tryptophan (Shiferaw Terefe and Augustin, 2020). The proposed process presented in our work with the use of soybean molasses can be considered as a “case study” since it cannot be compared with the current literature; indeed, no studies have investigated so far the use of this raw material as a natural source of tryptophan.

Another important distinguishing factor for the process we proposed is the high protein content in the biomass obtained at the end of the process. As KYNA biosynthesis is associated with tryptophan degradation, decrease in biomass at the end of the process was not surprising. Furthermore, exhaustion of the carbon source from the medium also decreases the protein content in the biomass. The proposed process using soybean molasses has advantages in two respects. First, tryptophan supply is assured causing an increased amount of KYNA biosynthesis, and second, the protein content in the yeast biomass did not decrease significantly (Figure 6).

Yeast used proteins, lipids, and some of the carbohydrates presented in this raw material as carbon and nitrogen sources. As a result, the yeast biomass was rich in both KYNA and proteins with a simultaneously low concentration of intracellular lipids. In fact, *Y. lipolytica* yeast has gained the GRAS status (generally recognize as safe) by the US Food and Drug Administration (Rywińska et al., 2013), and the biomass of *Y. lipolytica* is recognized as a novel food, according to the European Feed Manufacturers’ Federation (00575-EN). The protein content of the yeast biomass, the main by-products of the process, was analyzed using the Kjeldahl method (Kjeldahl, 1883). The highest protein concentration was observed for the *Y. lipolytica* A-101.131 GUT 1/8 strain (32%) growing on crude glycerol and the *Y. lipolytica* A-101.1.31 GUT1/6 mutant (35.6%) growing on crude glycerol and soybean molasses. The protein content recommended for fodder yeasts in the standards established by the FAO/WHO is 40%–52% (Juszczyk et al., 2013). The highest content of protein was observed in the post-culture biomass of *Y. lipolytica* (42.8%) cultured for erythritol production in the continuous mode using the glycerol-based medium and ammonium sulfate as a nitrogen source (Rakicka et al., 2016). Due to this fact, the residual biomass after the KYNA production in the process we proposed here may be used as a supplement for animal feeding. According to EU Regulation (EC) No 1829/2003, the application of genetically engineered biomass in food and feed is possible after authorization by the European Food Safety Authority. The yeast biomass requires the absence of viable cells, according to the European Food Safety Authority Panel on Nutrition, and then can be recognized as a novel food (Turck et al., 2019). In the presented work, non-pathogenic *Y. lipolytica* has been authorized on the market as a novel food intended for human nutrition by the Commission Implementing Regulation (EU) 2019/760.

The huge difference in KYNA production among the analyzed *Y. lipolytica* transformants was quite surprising; however, they could be the result of the site of integration of the expression cassette of *GUT1* gene as it was surrounded by the partial ZETA sequences. The random chromosomal integration could result in deletion of the gene being important for the regulation of tryptophan degradation or for downstream processing of KYNA. From the genome walking analysis, it turned out that two copies of the *GUT1* expression cassette were inserted into the genome of the *Y. lipolytica* A101-1.31 GUT1/1 strain both at the same arm of the *Y. lipolytica* E chromosome. One of the disrupted genes encodes fatty-acyl coenzyme A oxidase, an enzyme important for the beta oxidation process in this yeast. Deletion of this gene was already investigated during studies on polyhydroxyalkanoate biosynthesis in *Y. lipolytica*, and no information about KYNA biosynthesis could be found in the literature (Haddouche et al., 2010). The second disrupted gene encodes a protein with some similarities to *AUT1* from *S. cerevisiae*, which is essential for autophagocytosis. It is possible that the protein turnover inside the cell was somehow affected and constituted for additional supply in tryptophan, which could be further metabolized into KYNA. This hypothesis is being currently verified in another project.

Summarizing, the main product of this work, the dry biomass of yeast is rich in KYNA and protein. Moreover, it contains low amount of lipids. It was recently described that KYNA (5 mg kg^−1^ day^−1^) isolated from horseshoe crab inhibited both the body weight gain and the increase of average daily energy intake in high-fat diet–mice (Li et al., 2021). Additionally, KYNA reduced serum triglyceride and increased serum high-density lipoprotein cholesterol (Li et al., 2021). The functional food was defined by Granato et al. (2017) as industrially processed or natural food that when regularly consumed within a diverse diet at efficacious levels has potentially positive effects on health beyond basic nutrition. Yeast biomass obtained in this work fits into this definition and can be a healthy compound of the animal and human food. However, further research is necessary, especially metabolism engineering in the best producing strain to improve its assimilation and processing of the soybean molasses carbohydrates, which may serve as additional carbon sources. In the future studies, further genetic engineering of the best KYNA producer will be performed to enhance its biosynthesis. The crucial genes of tryptophan catabolism will be overexpressed—kynurenine formidase (BNA7) and kynurenine aminotransferase (KAT). Furthermore, deletion of genes encoding enzymes of alternative kynurenine pathway metabolism will be performed, to increase its amount available for KYNA production. Moreover, a large amount of work will also be focused on optimization of the type of culture. As mentioned earlier, we focused on batch and fed-batch cultures, which do not fully utilize the production potential of the cells. In the future work, a continuous process of kynurenic acid production by an industrially relevant strain will be investigated. Simultaneously, searching for another raw material, rich in tryptophan, will eliminate the need to supplement this amino acid.

## Conclusion

The obtained results showed that *Y. lipolytica* GUT1 mutants are able to produce KYNA in higher concentrations than the parental strain in a medium with crude glycerol. The results of this study represent a good starting point for further engineering of the *Y. lipolytica* A 101.1.31 GUT1/1 strain. Moreover, supplementation of the production medium with soybean molasses, the natural source of tryptophan, further increased the concentration of KYNA for A 101-1.31 wild strain and for the transformant A 101.1.31 GUT 1/1 strain. Our findings show the potential of *Y. lipolytica* producing KYNA and the post culture biomass as a promising ingredient of functional food for the treatment of obesity and hyperlipidemia, as well as for the modulation of gut microbiota in animals and humans.

## Data Availability

The original contributions presented in the study are included in the article/supplementary material; further inquiries can be directed to the corresponding author.
